# Draft genome sequence of CTX-M-5 variant-carrying *Kluyvera ascorbata* isolated from river water in Kumamoto, Japan

**DOI:** 10.1128/mra.01511-25

**Published:** 2026-03-13

**Authors:** Takatoshi Yamamoto, Chie Shitada, Tatsuya Kawaguchi, Shinji Narahara, Makoto Kuroda

**Affiliations:** 1 Department of Medical Technology, Faculty of Health Sciences, Kumamoto Health Science University 50183, Kumamoto, Japan; 2 Toxin and Biologicals Research Laboratory, Kumamoto Health Science University 50183, Kumamoto, Japan; 3 The Chemo-Sero-Therapeutic Research Institute (KAKETSUKEN) 13336, Kumamoto, Japan; University of Pittsburgh School of Medicine, Pittsburgh, Pennsylvania, USA

**Keywords:** *Kluyvera*, CTX-M, ESBL

## Abstract

The genus *Kluyvera* is presumed to be the original source of *bla*_CTX-M_ genes, which encode one of the most prevalent extended-spectrum β-lactamases. We report the draft genome sequence of *Kluyvera ascorbata* strain KHSU-R5-7, a CTX-M-5 variant producing isolate obtained from Shirakawa River in Kumamoto, Japan.

## ANNOUNCEMENT

**Fig 1:**
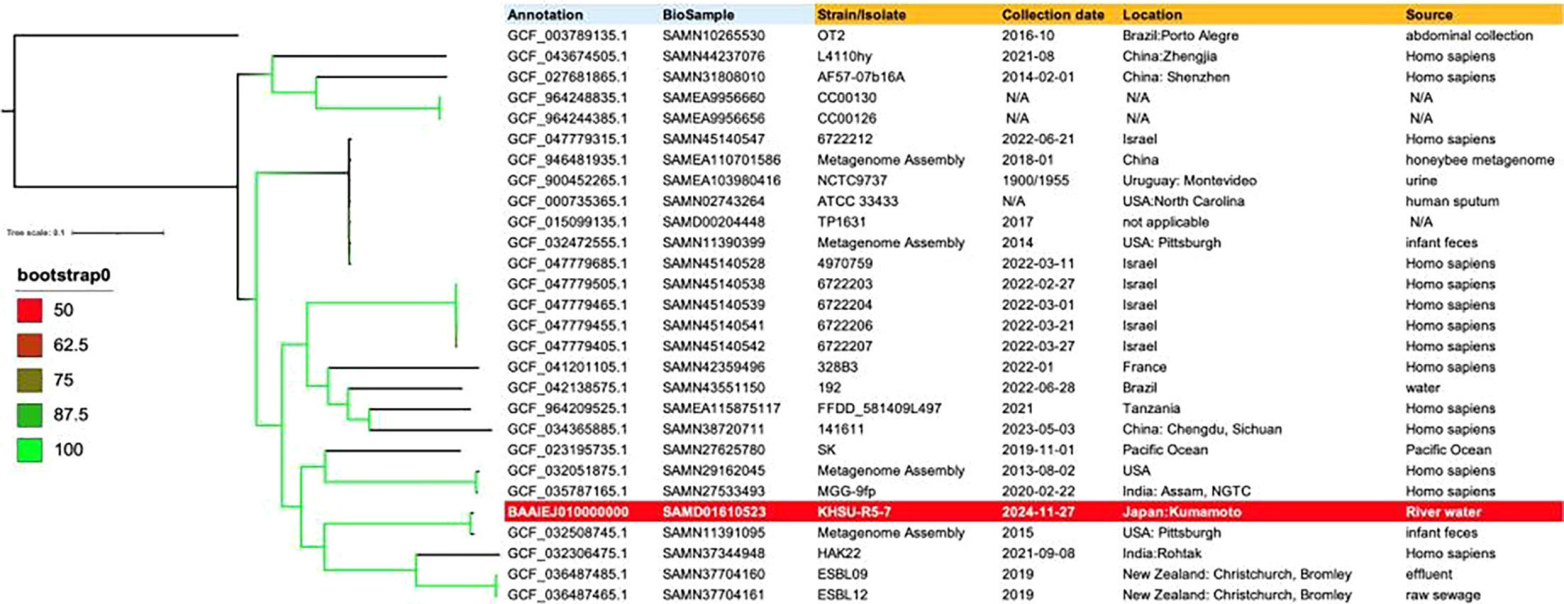
Core-genome SNV-based phylogenetic analysis of *Kluyvera ascorbata* strain KHSU-R5-7. A maximum-likelihood tree was reconstructed using 27 representative *K. ascorbata* strains. Whole-genome sequences were retrieved from the NCBI Datasets database (https://www.ncbi.nlm.nih.gov/datasets/genome/?taxon=51288). The complete genome of *K. ascorbata* TP1631 was used as the reference. The scale bar represents the number of substitutions per site. Bootstrap values (%) based on 1,000 replicates are indicated by the color-coded branches.

*Kluyvera ascorbata* is a gram-negative, catalase-positive, oxidase-negative, motile rod-shaped bacterium with peritrichous flagella. It belongs to the family Enterobacteriaceae (1). *K. ascorbata* can cause various opportunistic infections, including bacteremia and urinary tract infections (2). Furthermore, *K. ascorbata* is presumed to be the progenitor of *bla*_CTX-M_ genes, which encode some of the most widely disseminated extended-spectrum β-lactamases (ESBLs) (3). In this study, we isolated *K. ascorbata* strain KHSU-R5-7, which carries a *bla*_CTX-M-5_ variant, from river water in Kumamoto, Japan. Water samples were collected on 27 November 2024, and the strain was selected on CHROMagar ESBL plates after incubation at 35°C for 18 h. Genomic DNA was extracted from these cultures using the QIAGEN DNA extraction kit. Whole-genome sequencing was performed using the QIASeq FX DNA library kit (QIAGEN, Hilden, Germany) on the Illumina MiSeq platform with the MiSeq Reagent Kit v2 (250-bp paired-end reads). Additionally, a sequencing library was prepared using the Ligation Sequencing gDNA-Native Barcoding Kit 24 V14 (Oxford Nanopore Technologies, Oxford, UK). Sequencing was performed on a MinION Mk1b using an R10.4.1 flow cell controlled by MinKNOW v24.06.16, with basecalling carried out using Dorado v7.4.14. The Nanopore read N50 was 6,669 bp. Hybrid assembly of the Illumina and Nanopore reads using Unicycler v0.4.8 (4) yielded 14 unitigs with a total length of 5,448,488 bp (unitig N50: 3,716,739 bp; GC content: 53.9%). The genome contains 5,170 CDSs, 25 rRNAs, and 87 tRNAs, with a coding ratio of 87.3%. Sequence coverage was 75× for Illumina and 153× for Nanopore. Six plasmids were assembled into circular structures (ranging from 1,916 to 264,649 bp). However, the chromosome remained in two large unitigs (3.7 Mb and 1.4 Mb) due to a 29,889-bp repetitive sequence encoding phage tail, assembly, and terminase proteins; thus, a completely closed chromosome was not obtained. Genome annotation and taxonomic verification, including average nucleotide identity (ANI) calculations, were performed using DFAST v1.6.0 (5). Strain KHSU-R5-7 was identified as *K. ascorbata*, sharing 98.5% ANI with the reference strain ATCC 33433. ResFinder 4.6.0 identified a chromosomal *bla*_CTX-M-5_ (locus KHSUR5_32890) with a single nonsynonymous substitution (R7C) compared to KLUA-2 in *K. ascorbata* strain IP15.79. No mobile genetic elements were identified in its vicinity. Antimicrobial susceptibility testing using the microdilution method (Dry Plate 41, EIKEN CHEMICAL, Tokyo, Japan) following CLSI guidelines showed that strain KHSU-R5-7 is resistant to ampicillin, piperacillin, cefazolin, ceftriaxone, cefpodoxime, cefotaxime, cefotiam, cefepime, minocycline, and sulfamethoxazole/trimethoprim. Conversely, it remained susceptible to amikacin, meropenem, ceftazidime, cefmetazole, and levofloxacin. ESBL activity was confirmed by disk diffusion with cefotaxime-clavulanate. Notably, the susceptibility to ceftazidime is consistent with the characteristically low catalytic activity of the original CTX-M-5 toward this agent. Core-genome single-nucleotide variant (SNV) phylogenetic analysis was performed using 27 representative *K. ascorbata* strains with parsnp v1.7.4 (6), IQ-TREE2 v2.2.5 (7), and iTOL (8). The analysis revealed that KHSU-R5-7 is nearly identical to a strain (GCF_032508745.1) isolated from infant feces in Pittsburgh, USA, in 2015 (Fig. 1). This suggests that this lineage is not endemic to Japan but may be globally disseminated, warranting further surveillance.

## Data Availability

The whole-genome sequence has been deposited in DDBJ under the accession numbers BAAIEJ010000001 to BAAIEJ010000014 and BioSample accession number SAMD01610523. The Illumina and Nanopore sequence reads have been deposited in the DDBJ Sequence Read Archive (DRA) database under the accession numbers DRR719954 and DRR719955, respectively.
